# Relation of Perivascular Adipose Tissues on Computed Tomography to Coronary Vasospasm

**DOI:** 10.31083/RCM26327

**Published:** 2025-02-18

**Authors:** Kazunari Asada, Yuichi Saito, Hiroyuki Takaoka, Hideki Kitahara, Yoshio Kobayashi

**Affiliations:** ^1^Department of Cardiovascular Medicine, Chiba University Graduate School of Medicine, 260-8677 Chiba, Japan

**Keywords:** computed tomography, acetylcholine, vasospastic angina, inflammation

## Abstract

**Background:**

Coronary computed tomography angiography (CTA) can be used to quantitatively and qualitatively evaluate the characteristics of perivascular adipose tissue (PVAT), including PVAT volume and perivascular fat attenuation index (FAI). Moreover, PVAT volume and perivascular FAI on CTA are reportedly high in patients with vasospastic angina (VSA); however, previous investigations have focused on the patient rather than vessel-level analyses. Therefore, this study aimed to assess the relationship between coronary vasospasm and PVAT or FAI by using coronary CTA at the vessel level.

**Methods:**

This retrospective study included 51 patients who underwent intracoronary acetylcholine (ACh) provocation testing for the VSA diagnosis and coronary CTA within a 6-month interval. A total of 125 coronary vessels were evaluated. PVAT and FAI on CTA were quantitatively evaluated. The primary interest of the present study was to determine the relationship between PVAT volume and FAI- and ACh-induced coronary vasospasms at the vessel level.

**Results:**

Of the 51 patients, 24 (47.1%) had a positive ACh provocation test (VSA), with 40 of 125 (32.0%) vessels having ACh-induced vasospasm. Obstructive epicardial coronary artery disease was observed in 12 vessels (9.6%). No significant differences in PVAT volume or FAI were identified between vessels with and without ACh-induced vasospasms. Similarly, PVAT volume and FAI did not differ significantly in the individual major coronary arteries between patients with and without positive ACh provocation test results. In contrast, FAI was significantly higher in vessels with obstructive coronary artery disease than in those without.

**Conclusions:**

In patients undergoing intracoronary ACh provocation tests and coronary CTA, no significant association was observed between ACh-induced coronary vasospasm and PVAT volume or FAI at the vessel level. However, FAI significantly increased in vessels with epicardial coronary disease.

## 1. Introduction

Ischemia with nonobstructive coronary artery disease (CAD) has recently garnered significant recognition as a crucial subset of ischemic heart disease, among which vasospastic angina (VSA) is a major etiology [1]. In patients with suspected VSA, intracoronary acetylcholine (ACh) provocation testing is the gold standard invasive procedure [1, 2]. Previous studies have identified several clinical factors associated with VSA, including genetic variants, current smoking habits, and others [3, 4, 5]. From a mechanistic perspective, coronary vasospasm may be associated with the presence of perivascular adipose tissue (PVAT) and adventitial vasa vasorum as sources of inflammatory conditions [6]. Japanese researchers have confirmed that patients with VSA have significantly increased fluorodeoxyglucose uptake on positron emission tomography (PET) and PVAT volume on computed tomography (CT) surrounding the coronary arteries compared to those without VSA [6]. Recently, coronary artery inflammation has been evaluated by coronary CT angiography (CTA) using the fat attenuation index (FAI), a novel imaging biomarker that maps spatial changes in perivascular fat attenuation. This biomarker is reportedly associated with future cardiovascular outcomes in patients with obstructive and non-obstructive coronary diseases [7, 8]. Some previous studies have indicated a potential relationship between such findings on coronary CTA and vasospasm, however, most of these studies focused on patients with VSA (i.e., patient-level analysis) rather than coronary arteries with vasospasm (i.e., vessel-level analysis) [9, 10]. Thus, we aimed to assess the characteristics of PVAT on CTA of coronary arteries with vasospasms in patients undergoing ACh provocation tests.

## 2. Materials and Methods

### 2.1 Study Population

**Fig. 1.**
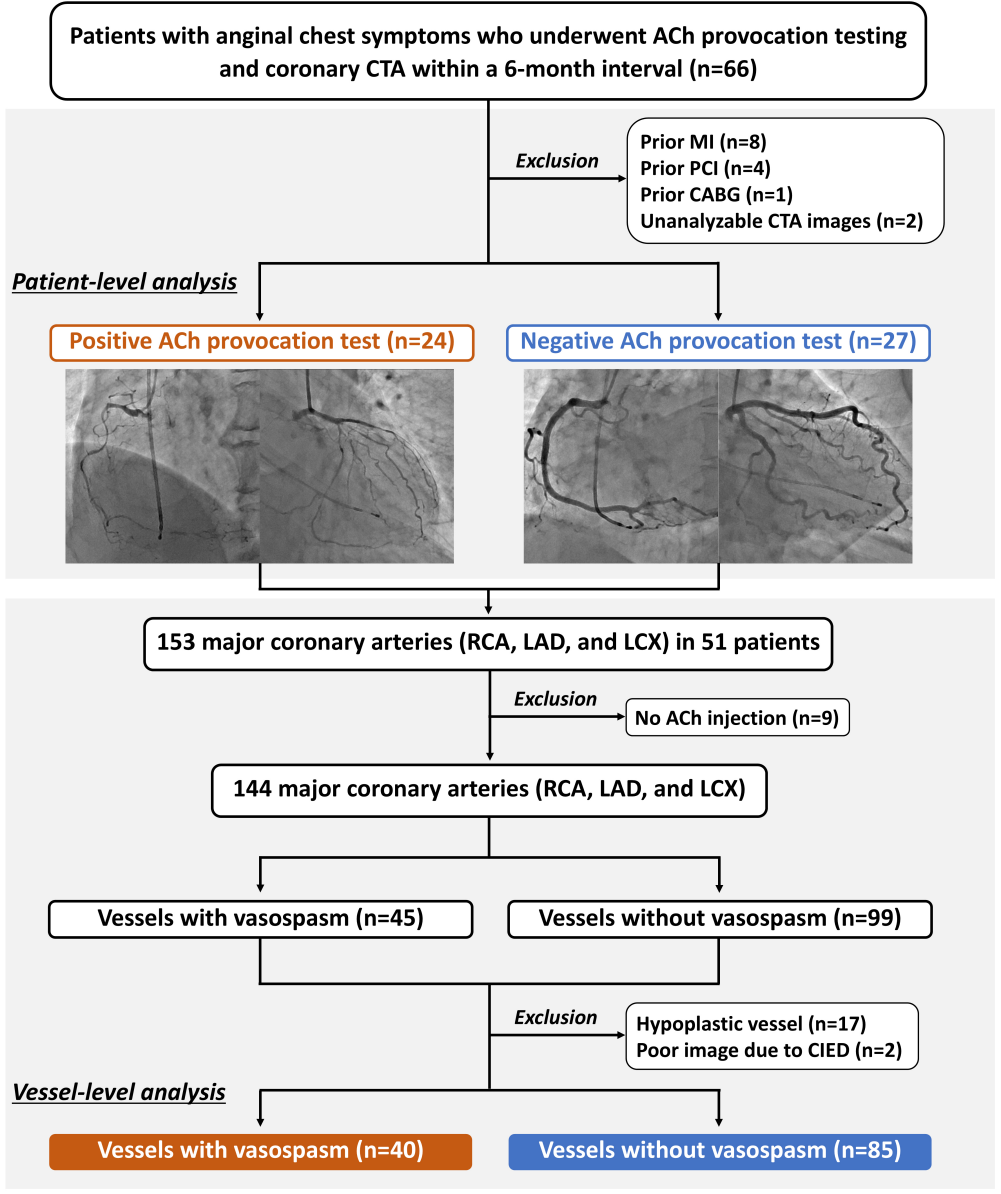
** Study flow**. ACh, acetylcholine; CABG, coronary artery bypass grafting; CIED, cardiac implantable electronic device; CTA, computed tomography angiography; LAD, left anterior descending; LCX, left circumflex; MI, myocardial infarction; PCI, percutaneous coronary intervention; RCA, right coronary artery.

This retrospective observational study was conducted at the Chiba University Hospital. Between May 2012 and June 2023, 66 patients presenting with anginal chest symptoms underwent intracoronary ACh provocation testing for the diagnosis of VSA and coronary CTA within a 6-month interval. Patients with prior myocardial infarction (n = 8), percutaneous coronary intervention (n = 4), coronary artery bypass grafting (n = 1), or unanalyzable CTA images (n = 2) were excluded. Finally, 51 patients were included in the analysis (Fig. 1). Among the three main coronary arteries, namely the left anterior descending (LAD), left circumflex (LCX), and right coronary artery (RCA), a total of 153 vessels were assessed in the included patients. Vessels were excluded if intracoronary ACh was not administered (n = 9), if they were hypoplastic (n = 17), or if they were unanalyzable due to the presence of cardiac implantable electronic devices (n = 2). Finally, 125 vessels were evaluated in the present study (Fig. 1). Informed consent was obtained in an opt-out manner. The present study was performed in accordance with the Declaration of Helsinki and was approved by the Ethics Committee of Chiba University Hospital.

### 2.2 ACh Provocation Test

Intracoronary ACh provocation testing was performed according to guideline recommendations [1], as previously described [11, 12, 13]. Briefly, vasodilating medications were discontinued ≥48 hours before the ACh tests in elective cases, except for short-acting sublingual nitroglycerin as needed. Coronary angiography was performed predominantly via the radial artery and brachial vein [14, 15]. After performing controlled angiography, a temporary pacing electrode was placed in the right ventricle to prevent bradycardia. ACh was administered in incremental doses of 20, 50, and 100 µg into the left coronary artery initially, followed by doses of 20 and 50 µg into the RCA, with each injection given over a 20-second period. Coronary vasospasm was assessed 1 minute after each ACh dose administration. Intracoronary isosorbide dinitrate (1–2 mg) was introduced into both coronary arteries, followed by coronary angiography. Obstructive epicardial CAD was identified as ≥50% stenosis observed on the post-isosorbide dinitrate angiogram. A positive ACh provocation test was defined as angiographic evidence of coronary artery vasospasm (total or subtotal occlusion) accompanied by chest symptoms and/or ischemic changes on electrocardiography (ECG). Additionally, ACh test results were independently evaluated by two experienced cardiologists who were blinded to the patient’s clinical characteristics.

### 2.3 Coronary CTA Protocol

Coronary CTA was performed using either a 320-detector (Aquilion ONE or Aquilion ONE ViSION Edition, Canon Medical Systems, Otawara, Japan) or a 256-detector (Revolution APEX, GE Healthcare, Waukesha, WI, USA) row CT system. The procedure was initiated with scout and non-contrast ECG-synchronized cardiac scans utilizing a prospective electrocardiogram gating method before contrast administration. The 320-row CT scans were performed with a 0.5 mm slice thickness and 80–120 kV tube voltage, whereas the 256-row CT scans utilized a 0.625 mm slice thickness and 70 kV tube voltage. To reduce radiation exposure during systolic phases while maintaining high diagnostic accuracy for detecting coronary artery stenosis, retrospective ECG gating with dose modulation was employed (0.5 mm slice thickness and 120 or 135 kV tube voltage for the 320-slice system and 0.625 mm slice thickness and 120 kV tube voltage for the 256-slice system). To evaluate epicardial CAD, the patients received sublingual nitrates immediately before the scan. When the heart rate exceeded 65 beats per minute (bpm), intravenous landiolol (12.5 mg) was administered before scanning unless contraindicated. The contrast protocol consisted of three phases: 40–100 mL of undiluted iodinated contrast agent (350–370 mg I/mL) at 3–5 mL/s; 0–50 mL of a 1:1 saline-contrast mixture at 3–4 mL/s; and 20–30 mL of saline at 2–4 mL/s).

### 2.4 Image Analysis

**Fig. 2.**
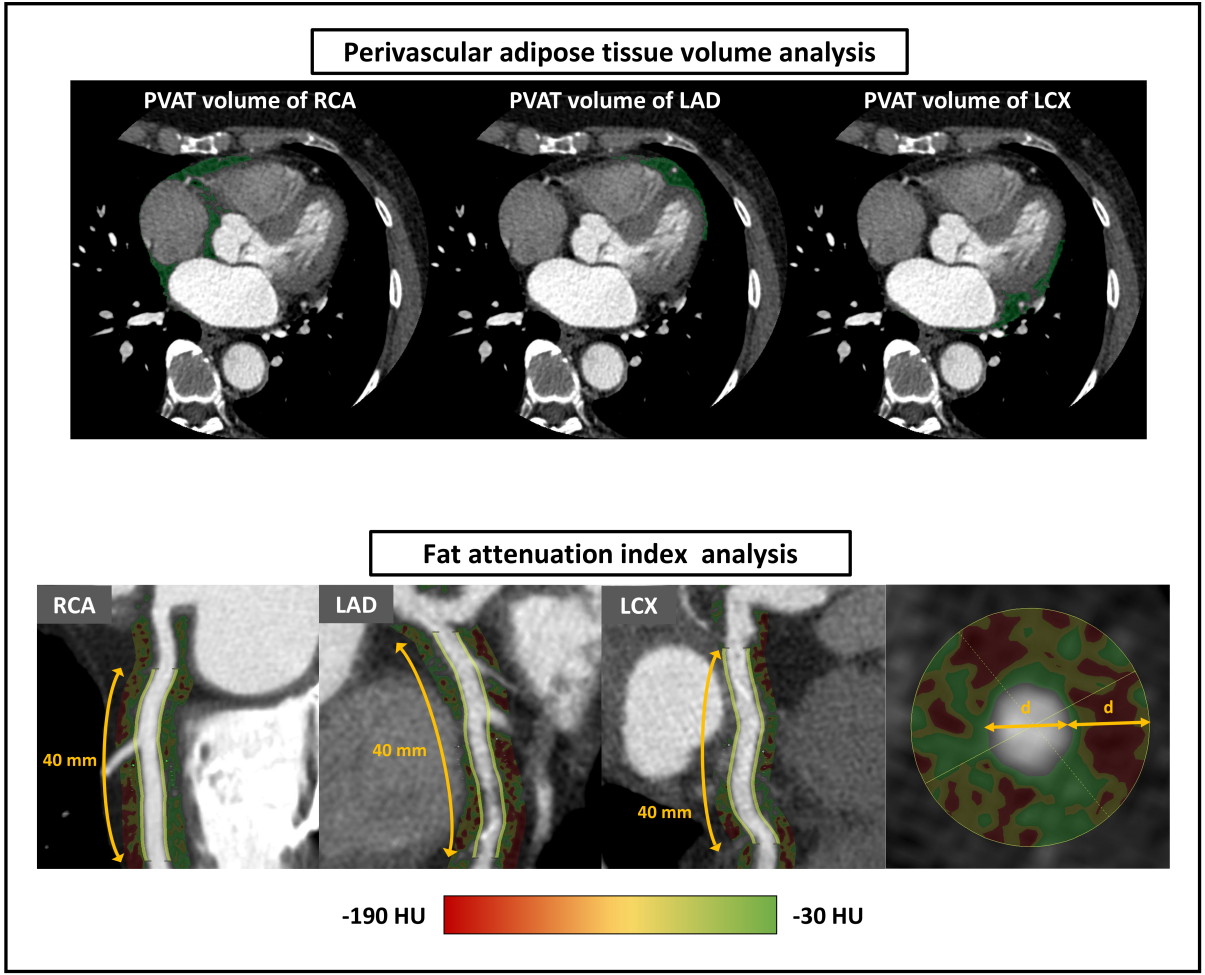
**Coronary computed tomography angiography (CTA) analysis for perivascular adipose tissue (PVAT) volume and fat attenuation index (FAI)**. The PVAT is shown as a green area on CTA. Furthermore, PVAT was identified as the tissue surrounding each main coronary artery with a density ranging from –195 to –45 HU from the right pulmonary artery down to the diaphragm. Fig. 2 displays perivascular FAI as the area of the color gradient. Perivascular FAI is defined as the mean CT attenuation of perivascular tissue with a density ranging from –190 to –30 HU measured within a radial distance equal to the vessel diameter. This is applied to the most proximal segment of the coronary arteries for 40 mm in the LAD and LCX, and the segment from 10 to 50 mm from the ostium in the RCA. CT, computed tomography; HU, Hounsfield unit; LAD, left anterior descending; LCX, left circumflex; RCA, right coronary artery.

Epicardial adipose tissue (EAT) is a fat deposit located between the surface of the heart and the visceral layer [16]. Using dedicated software (SYNAPSE VINCENT, FUJIFILM, Tokyo, Japan), EAT was identified as tissue with a density ranging from –195 to –45 Hounsfield units (HU) [16]. To calculate the EAT volume, EAT areas were measured at 1.0 cm intervals from the level of the right pulmonary artery down to the diaphragm, and these measurements were then totalled [17]. PVAT areas were defined as fat deposits surrounding the main coronary arteries: the LAD, LCX, and RCA (Fig. 2). If the PVAT overlapped between the LAD and either the RCA or LCX, the area of interest was extended to the midpoints of these regions [18]. The volume of the coronary PVAT was determined using a method similar to that used for the EAT volume calculation. The volumes of the EAT and coronary PVAT were indexed using body surface area (BSA). FAI was analyzed using the same software (SYNAPSE VINCENT). Perivascular FAI was defined as the mean CT attenuation of PVAT between –190 and –30 HU [19]. Measurements were obtained within a radial distance from the outer vessel wall equal to the diameter of the respective vessel surrounding the coronary arteries. According to a previous report, FAI was evaluated in the most proximal segment of the coronary arteries at 40 mm in the LAD and LCX [19], whereas the segment from 10 to 50 mm from the ostium was analyzed in the RCA to avoid the effects of the aortic wall (Fig. 2). The left main coronary artery was not assessed.

### 2.5 Outcomes and Statistical Analysis

The primary interest of the present study was to determine the relationship between PVAT volume and FAI and ACh-induced coronary vasospasms at the vessel level. Secondary outcomes included the PVAT volume and FAI in patients with positive and negative ACh provocation test results. Statistical analyses were performed using EZR (Saitama Medical Center, Jichi Medical University, Saitama, Japan), a graphical user interface for R (R Foundation for Statistical Computing, Vienna, Austria). Continuous variables are expressed as mean ± standard deviation or median with interquartile range, meanwhile, categorical variables are demonstrated as frequencies with percentages. Continuous variables were compared using Student’s *t*-test or the Mann–Whitney U test, whereas categorical variables were evaluated using Fisher’s exact test. Statistical significance was set at *p*-value < 0.05.

## 3. Results

Of the 51 patients, 24 (47.1%) yielded a positive ACh provocation test (VSA), with 40 of the 125 (32.0%) vessels having significant ACh-induced vasospasm (Fig. 1). Obstructive epicardial CAD was observed in 12 (9.6%) vessels. The baseline characteristics did not differ significantly between patients with or without VSA, except for BSA (Table 1). The findings of the ACh provocation tests are presented in Table 2. In patients with vasospasms in the LAD and RCA in the negative ACh test group (Table 2), neither chest symptoms nor ECG changes were observed.

**Table 1.** **Baseline characteristics**.

Variable		Positive ACh test	Negative ACh test	*p*-value
		(n = 24)	(n = 27)	
Age (years)		61.2 ± 11.4	60.1 ± 16.8	0.78
Men		11 (45.8%)	17 (63.0%)	0.27
Body surface area (m^2^)		1.61 ± 0.16	1.75 ± 0.27	0.03
Hypertension		15 (62.5%)	17 (63.0%)	1.00
Diabetes		2 (8.3%)	7 (25.9%)	0.15
Dyslipidemia		15 (62.5%)	16 (59.3%)	1.00
Current smoker		3 (12.5%)	4 (14.8%)	1.00
eGFR (mL/min/1.73 m^2^)		76.7 ± 19.1	73.1 ± 20.1	0.52
HbA1c (%)		5.8 ± 0.4	5.9 ± 0.7	0.54
HDL-C (mg/dL)		70.9 ± 28.5	61.6 ± 17.9	0.17
LDL-C (mg/dL)		124.0 ± 40.8	116.2 ± 27.4	0.43
Triglyceride (mg/dL)		123.4 ± 72.0	145.5 ± 94.0	0.36
BNP (pg/mL)		32.2 (9.5, 50.9)	19.0 (11.8, 77.0)	0.84
LVEF (%)		61.6 ± 9.2	61.5 ± 9.7	0.99
Medical treatment				
	Calcium channel blocker	11 (45.8%)	14 (51.9%)	0.78
	Long-acting nitrate	3 (12.5%)	3 (11.1%)	1.00
	Nicorandil	2 (8.3%)	4 (14.8%)	0.67
	Antiplatelet	3 (12.5%)	4 (14.8%)	1.00
	Statin	8 (33.3%)	9 (33.3%)	1.00
	ACE-I, ARB, or ARNI	10 (41.7%)	8 (29.6%)	0.40
	β-blocker	4 (16.7%)	8 (29.6%)	0.34

ACE-I, angiotensin-converting enzyme inhibitor; ACh, acetylcholine; ARB, angiotensin II receptor blocker; ARNI, angiotensin receptor-neprilysin inhibitor; BNP, B-type natriuretic peptide; eGFR, estimated glomerular filtration rate; HbA1c, hemoglobin A1c; HDL-C, high-density lipoprotein cholesterol; LDL-C, low-density lipoprotein cholesterol; LVEF, left ventricular ejection fraction.

**Table 2.** **Acetylcholine provocation test findings**.

Variable		Positive ACh test	Negative ACh test	*p*-value
		(n = 24)	(n = 27)	
Obstructive epicardial CAD		6 (25.0%)	6 (22.2%)	1.00
ACh provocation in the LCA		24 (100%)	27 (100%)	1.00
ACh provocation in the RCA		17 (70.8%)	25 (92.6%)	0.07
Number of spasm vessels		2 [1, 2]	0 [0, 0]	<0.001
	Vasospasm of the LAD	19 (79.2%)	3 (11.1%)	<0.001
	Vasospasm of the LCX	12 (50.0%)	0 (0.0%)	<0.001
	Vasospasm of the RCA	9 (52.9%)	2 (8.0%)	0.003
Multivessel spasm		13 (54.2%)	0 (0.0%)	<0.001
Sings of ischemia				
	Chest symptoms	19 (79.2%)	8 (29.6%)	<0.001
	ECG changes	21 (87.5%)	7 (25.9%)	<0.001

ACh, acetylcholine; CAD, coronary artery disease; ECG, electrocardiography; LAD, left anterior descending; LCA, left coronary artery; LCX, left circumflex; RCA, right coronary artery.

**Fig. 3.**
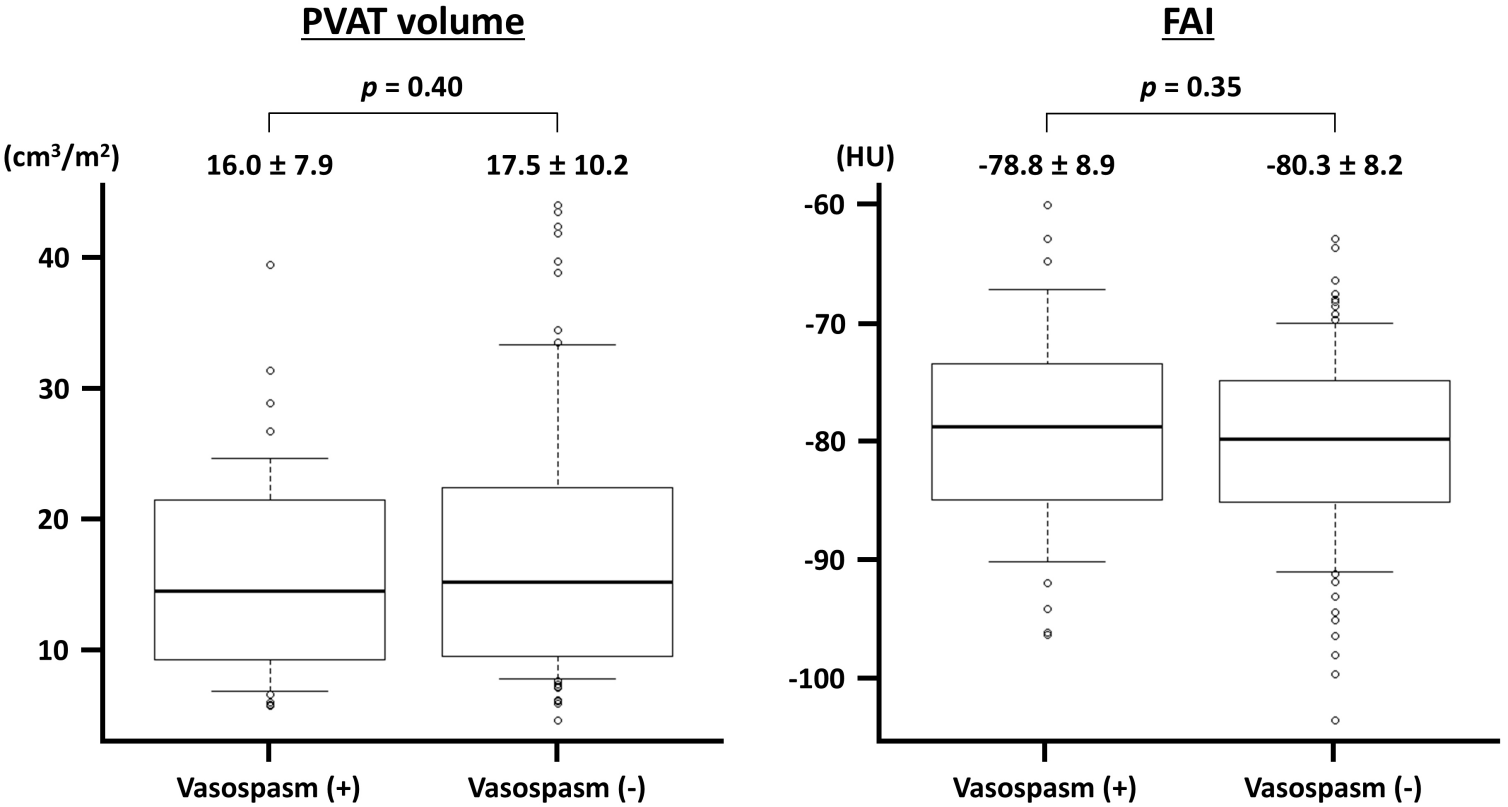
**Perivascular adipose tissue (PVAT) volume and fat attenuation index (FAI) in vessels with and without acetylcholine-induced vasospasm**. The PVAT volume is indexed to the body surface area. HU, Hounsfield unit.

**Fig. 4.**
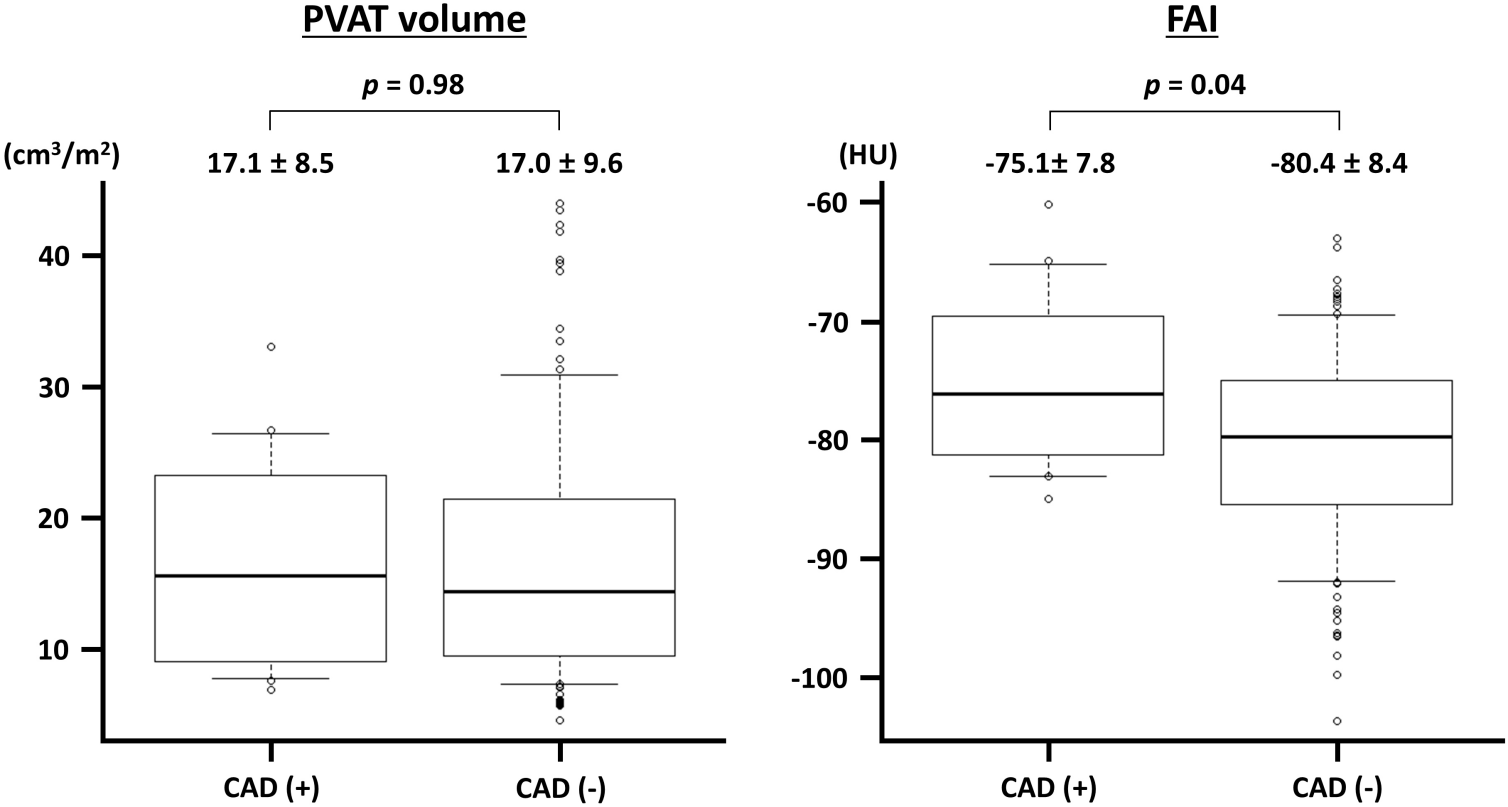
**Perivascular adipose tissue (PVAT) volume and fat attenuation index (FAI) in vessels with and without obstructive coronary artery disease (CAD)**. The PVAT volume is indexed to the body surface area. HU, Hounsfield unit.

Overall, volumes of EAT and PVAT were 84.9 ± 41.2 and 28.3 ± 17.2 cm^3^, respectively, while perivascular FAI was –79.8 ± 8.4. In the vessel-level analysis, the BSA-indexed volume of the PVAT was not significantly different between the vessels with (n = 40) and without (n = 85) ACh-induced vasospasms (Fig. 3). Similarly, no significant difference was identified in FAI between the two groups (Fig. 3). In each major coronary artery, the presence of ACh-induced vasospasm was not significantly associated with the PVAT volume or FAI (Table 3). Conversely, vessels with epicardial obstructive CAD had a significantly higher FAI than those without (Fig. 4). In the patient-level analysis, the BSA-indexed volumes of the PVAT and FAI did not differ significantly between patients with and without positive ACh provocation test results in the LAD, LCX, and RCA (Table 4).

**Table 3.** **Vessel-level coronary computed tomography angiography analysis in each artery**.

Variable		ACh-induced vasospasm (+)	ACh-induced vasospasm (-)	*p*-value
PVAT volume (cm^3^/m^2^)				
	LAD	14.7 ± 6.6 (n = 20)	15.4 ± 10.0 (n = 27)	0.81
	LCX	12.1 ± 6.8 (n = 9)	11.9 ± 5.0 (n = 28)	0.92
	RCA	21.4 ± 8.6 (n = 11)	24.7 ± 9.8 (n = 30)	0.32
Perivascular FAI (HU)				
	LAD	–80.4 ± 8.7 (n = 20)	–80.8 ± 6.3 (n = 27)	0.83
	LCX	–75.2 ± 7.2 (n = 9)	–77.4 ± 6.4 (n = 28)	0.38
	RCA	–78.9 ± 10.4 (n = 11)	–82.6 ± 10.5 (n = 30)	0.33

The PVAT volume was indexed to body surface area. ACh, acetylcholine; FAI, fat attenuation index; HU, Hounsfield unit; LAD, left anterior descending; LCX, left circumflex; PVAT, perivascular adipose tissue; RCA, right coronary artery.

**Table 4.** **Patient-level coronary computed tomography angiography analysis**.

Variable		Positive ACh test	Negative ACh test	*p*-value
		(n = 24)	(n = 27)	
PVAT volume (cm^3^/m^2^)				
	LAD	13.9 ± 6.9	16.0 ± 9.5	0.37
	LCX	11.1 ± 5.2	12.2 ± 5.4	0.46
	RCA	21.8 ± 9.2	24.6 ± 10.6	0.33
Perivascular FAI (HU)				
	LAD	–81.1 ± 8.3	–80.9 ± 6.5	0.91
	LCX	–76.5 ± 7.0	–77.7 ± 6.9	0.56
	RCA	–82.5 ± 10.4	–81.0 ± 9.8	0.59

The PVAT volume was indexed to body surface area. ACh, acetylcholine; FAI, fat attenuation index; HU, Hounsfield unit; LAD, left anterior descending; LCX, left circumflex; PVAT, perivascular adipose tissue; RCA, right coronary artery.

## 4. Discussion

The present study demonstrated that PVAT analysis using CTA was feasible in 125 of 144 (86.8%) coronary vessels and that ACh-induced vasospasm was not significantly associated with increased PVAT volume and FAI at the vessel level. The presence of obstructive epicardial CAD was associated with elevated FAI. The present study findings suggest that the PVAT, but not the VSA, was involved in the potential pathogenesis of obstructive CAD.

Previous studies have identified several underlying mechanisms of coronary artery spasms, including inflammation, endothelial dysfunction, and smooth muscle cell hypercontractivity [20]. In this context, PVAT may play a crucial role as a source of inflammation. The PVAT functions as an active endocrine and paracrine organ that secretes inflammatory cytokines [21]. In an experimental study involving pigs, inflammatory changes in PVAT on PET images were associated with coronary hypercontractivity [22]. Additionally, previous clinical studies have demonstrated that fluorodeoxyglucose uptake on PET, a surrogate of inflammation, was significantly increased in patients with positive ACh provocation tests compared to the uptake in those without negative test results [6]. Consequently, an association between coronary spasm and inflammation of the coronary adventitia and PVAT was suggested. Furthermore, a correlation between increased PET/CT uptake and elevated CT attenuation values around the coronary arteries has been reported [23]. Previous studies have investigated the clinical relevance of the pericoronary adipose tissue in a broad spectrum of patient populations [24, 25]. CTA is another non-invasive modality used to evaluate PVAT and inflammation. Although PVAT assessed by CTA has been associated with future cardiovascular risk in previous studies [17, 26], its prognostic significance remains to be established. In the CRISP-CT study, another CT measure of PVAT, namely FAI, was evaluated, which enhanced cardiac risk stratification in patients with mild-to-moderate epicardial CAD [7]. The subsequent ORFAN study demonstrated the clinical utility of FAI on coronary CTA for cardiovascular risk estimation in patients without obstructive CAD [8]. Although some previous studies have investigated the potential increase in PVAT and FAI on coronary CTA in patients with VSA [9, 10], the results were preliminary and focused only on patient-level analysis, preventing evaluation of a direct link between ACh-induced epicardial vasospasm and PVAT. Thus, in the present study, we primarily focused on a vessel-level analysis of the relationship between CTA metrics and vasospasm.

In this context, the present study investigated the impact of PVAT volume on FAI. In a previous study in Japan by Ohyama *et al*. [10], which included 66 patients who underwent ACh provocation tests and coronary CTA, the BSA-indexed PVAT volume ranged from approximately 7 to 20 cm^3^/m^2^ in each major coronary artery. These findings potentially align with our results [10]. As reported in the Japanese study, the PVAT volume was reduced in the LCX compared to the LAD and RCA in the present study. Ohyama *et al*. [10] demonstrated that the PVAT volume in the LAD on coronary CTA was significantly increased in patients with VSA compared to that in the control group. In the landmark CRISP-CT study, the mean FAI reportedly ranged from –75.1 to –77.0 HU in their derivation and validation cohorts [7], which also aligned with our study results. Another Japanese study indicated that FAI in the RCA could predict positive ACh provocation tests [9, 10]. Collectively, previous studies suggest that PVAT volume and FAI assessed on coronary CTA may be associated with positive results in ACh provocation tests. However, the key coronary arteries differed between the two metrics (LAD vs. RCA). In the present study, the vessel-level analysis did not demonstrate a significant association between ACh-induced vasospasm and PVAT volume or FAI. Although the underlying mechanisms of these insignificant results remain unclear, we believe that this association is neither simple nor robust. Importantly, the FAI was significantly higher in vessels with obstructive CAD in the present study, reinforcing the idea that inflammation enhances coronary atherosclerosis [27]. Further studies are needed to refine the methodology of CTA analysis and to identify patients with VSA that are highly likely to be attributable to PVAT and inflammation. Moreover, PET may be a superior diagnostic modality for assessing inflammation surrounding the coronary arteries [6].

Our study had some limitations that should be considered when interpreting the results. As this was a single-center retrospective study with a relatively small sample size, external generalizability may be limited, and potential selection bias should be acknowledged. Thus, our results should be considered hypothesis-generating. Although the CT images were analyzed following previously reported methods [22], these have not yet been fully established. In the present study, intracoronary ACh provocation testing and coronary CTA were performed within 6 months, during which the characteristics of PVAT possibly changed due to lifestyle modifications and medications. In the negative ACh test group, some patients presented with chest symptoms and ECG changes during provocation testing (Table 2), suggesting a potential diagnosis of microvascular spasm. However, the small sample size precluded further analyses of this patient population.

## 5. Conclusions

In patients undergoing intracoronary ACh provocation tests and coronary CTA, no significant association was noted between ACh-induced coronary vasospasm and PVAT volume or FAI at the vessel level, although FAI significantly increased in vessels with epicardial obstructive CAD. Future prospective, multicenter, well-designed investigations with large sample sizes are warranted to clarify the impact of PVAT quantity and quality on coronary vasospasm.

## Availability of Data and Materials

The datasets used and/or analyzed during the current study are available from the corresponding author on reasonable request.
